# A chain of survival for drug overdose

**DOI:** 10.1016/j.resplu.2025.101026

**Published:** 2025-07-08

**Authors:** Matthew Robert Dernbach, Nabarun Dasgupta, Adams L. Sibley, Ida Tylleskar, Arne Kristian Skulberg

**Affiliations:** a Department of Emergency Medicine, Emory University, Atlanta, GA, USA; b Injury Prevention Research Center, University of North Carolina, Chapel Hill, NC, USA; c Department of Circulation and Medical Imaging, Norwegian University of Science and Technology, Trondheim, Norway; d Department of Internal Medicine, St. Olavs Hospital, Trondheim University Hospital, Trondheim, Norway; e Division of Prehospital Services, Oslo University Hospital, Oslo, Norway

**Keywords:** Overdose, Opioids, Naloxone, Cardiac arrest, Chain of survival

## Abstract

Drug overdose is a leading global cause of preventable morbidity and mortality and is closely associated with out-of-hospital cardiac arrest. While the American Heart Association’s “chain of survival” has improved outcomes in traditional cardiac arrest, it does not address the unique characteristics and challenges of overdose-related emergencies. In this article, we propose a novel “overdose chain of survival” to emphasize the importance of linking acute care with long-term prevention. This six-link framework includes: (1) prevention, (2) recognition and activation of emergency services, (3) basic first aid, (4) emergency treatment, (5) post-overdose care, and (6) recovery and secondary prevention. Each link highlights opportunities for intervention and coordination across systems of care. The framework is intended to guide clinical care, policy development, education, and research. By viewing overdose through a structured and holistic lens, the overdose chain of survival seeks to improve outcomes in the immediate aftermath of an overdose and also to address systemic barriers to sustained recovery. This conceptual model is adaptable to evolving drug trends and highlights the need for integrated, patient-centered approaches to overdose response and prevention.

## Highlights

• Identifies gaps in the continuity of overdose care.
• Proposes a novel six-link “Overdose Chain of Survival” tailored to overdose-related emergencies.
• Emphasizes integration of acute overdose care with long-term recovery and prevention.

## Introduction

Substance use disorder and drug overdose, particularly from opioids, are leading causes of death globally and are increasingly prevalent in all regions in the world, contributing to the global burden of disease. This includes overdose-associated out-of-hospital cardiac arrest, of which opioid use is a significant risk factor.^1^, ^2^, ^3^, ^4^ The United States has suffered from several waves of an opioid overdose epidemic, most recently involving fentanyl and fentanyl analogs. In Europe, there is concern that nitazenes are increasingly replacing heroin in the illicit drug supply. In Africa and the Middle East, there is rising use of tramadol. And in Central Asia, heroin is a significant problem.^5^, ^6^

The mechanism of opioid-related death is central nervous system and respiratory depression, leading to cardiac arrest. Relative to other forms of cardiac arrest, overdose-related arrests tend to be younger, have non-shockable rhythms, and are more likely to survive.^7^, ^8^, ^9^ The etiology of an overdose is often multifactorial, related to the involved substances, as well as their potency and interactions. Vulnerability of the individual patient, the local drug supply, and upstream structural factors also contribute.^10^ Although management might differ based on the specific substance underlying an overdose, many of the tenets of antidotal therapy and symptomatic and supportive care remain applicable in all overdose-related scenarios.^11^, ^12^ The same is true for cardiac arrest, where both cardiac and non-cardiac causes are recognized as culprits, but the treatment recommendations and care pathway are similar in broad terms.^13^ In both overdose and cardiac arrest, focus on acute interventions, while important, can overshadow opportunities for primary and secondary prevention that have proven impacts on mortality reduction.

The chain of survival was conceptualized by Friedrich Wilhelm Ahnefeld and described in 1991 by the American Heart Association (AHA) as a framework to describe a particular sequence of events that, if implemented, can improve the outcomes of out-of-hospital sudden cardiac arrest.^14^, ^15^ The AHA currently describes four different chains of survival, differentiating between inside and outside of the hospital and adult and pediatric cases.^16^ The chains are updated to align with research and emerging trends, and have proven to be instrumental to cardiac arrest management as well as for policy development, research, and the teaching of individual first aid providers.^17^ Furthermore, the concept has been adapted to other clinical scenarios such as traumatic injury, among many others.^18^, ^19^

As drug overdose is a significant driver of morbidity and mortality and closely linked to cardiac arrest, we believe a separate chain of survival specifically tailored to overdose is called for. We have not performed a formal gap analysis or Delphi process in the work presented here. However, the current set of chains fail to capture the unique challenges facing overdose-associated cardiac arrest such as reluctance to alert emergency services, the wide presence of antidotes in the community, and the hesitancy to engage in further care after naloxone. Additional gaps in the process of overdose management are described elsewhere in the literature, including timely intervention in the acute phase as well as facilitating sustained engagement with healthcare and long-term psychosocial support post-overdose.^20^, ^21^ Naloxone is a cornerstone of opioid overdose reversal, but an over-emphasis on antidotal treatment during opioid-related emergencies has the potential to overshadow other vital elements.^11^ For instance, the way over-the-counter naloxone nasal spray labels were mandated by the United States Food and Drug Administration emphasized the pharmacological aspect of opioid overdose response while neglecting the importance of cardiopulmonary resuscitation (CPR), rescue breathing, and follow-up care.^22^ To fill these gaps, we propose applying the chain of survival framework for cardiac arrest to the management of overdose.

**Fig. 1:**
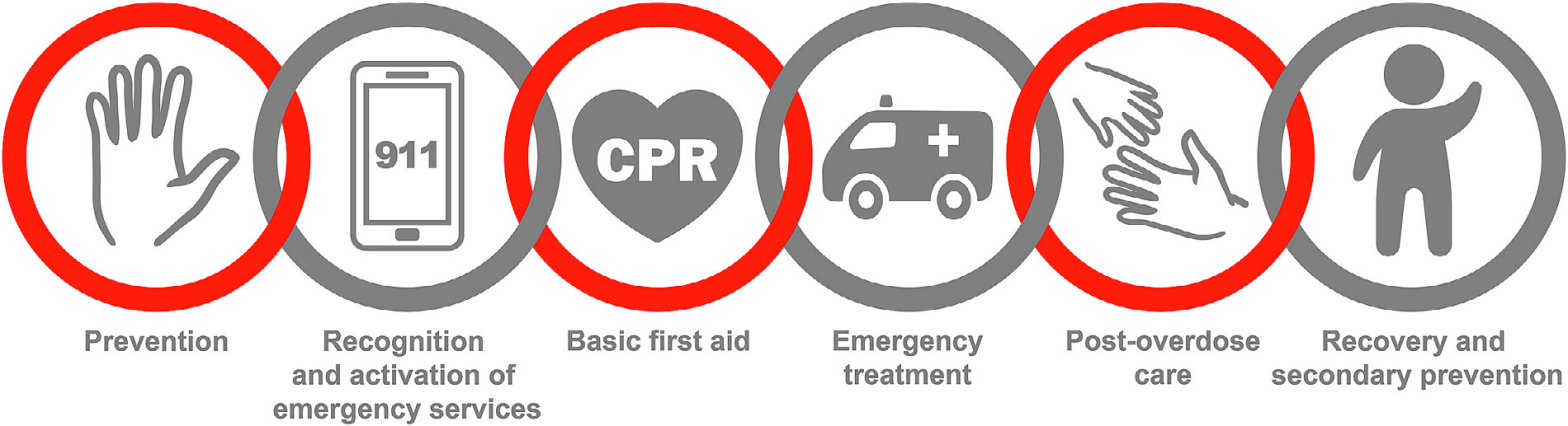
Proposed framework for the overdose chain of survival. (Illustration by Øystein Horgmo, Medical Photography and Illustration Service, University of Oslo).

We believe that an overdose chain of survival is applicable to life-threatening toxicity due to poisoning from any substance. We have chosen to focus on opioids because of their significant public health impact and association with cardiac arrest. We propose that the chain of survival schema can be adapted to overdose, to include: first, prevention; second, recognition and activation; third, basic first aid; fourth, emergency treatment; fifth, post-overdose care; and sixth, recovery and secondary prevention (Fig. 1). We have developed this framework for use by the general public, health care professionals, researchers, and policy makers.

## The overdose chain of survival

### Prevention

Primary prevention for overdose includes addressing the various root causes of substance use and other contributing factors to overdose. Relevant to opioid overdoses, these include individual social determinants, patient and clinician education regarding opioid use and prescribing, opioid-related research and funding, screening for conditions that increase risk for overdose (e.g., opioid use disorder), and promotion of harm reduction strategies.^10^, ^23^

### Recognition and activation

Bystanders must be able to recognize an overdose and activate the emergency response, which provides advice and ensures an ambulance can be sent. The elements of the emergency response may include multidisciplinary collaboration between emergency medical services (EMS), mental health services, poison centers, and community-based harm reduction services.

Vital to this link is improving layperson and professional awareness of overdose symptoms and reducing barriers to notifying emergency services. This would involve community education efforts about the signs and symptoms of overdose, with particular attention given to individuals likely to witness an opioid overdose. Public health efforts and policy changes must also focus on factors that may hinder bystanders from notifying emergency services, including fear of prosecution.^24^

### Basic first aid

The initial step in this process is stimulation of an unconscious person, with repositioning and freeing of the airway to enable and detect breathing. Because it can be challenging to determine pulselessness without medical equipment and training, the AHA recommends lay-rescuers to assume and treat for cardiac arrest by performing CPR on anyone who is unconscious and not breathing normally.^12^ The risk of harm by CPR to patients that are not in cardiac arrest is low, and the benefit of early chest compressions is high.^16^ Implementing this link requires community education efforts around CPR. This includes teaching and focus on this as a part of the naloxone response for laypeople.

Through naloxone distribution and education programs, which have been effectively implemented in countries of varying socioeconomic status, bystanders can administer naloxone prior to EMS arrival.^25^, ^26^, ^27^, ^28^ For laypeople, the administration of an antidote should not precede physical stimulation and other basic first aid measures or delay CPR.^12^ The products available for overdose reversal by laypeople must be designed to allow easy administration and the potential for titration.

### Emergency treatment

Emergency health care personnel must evaluate all patients who have experienced an overdose, as many may need further advanced life support. Even if a bystander has reversed an overdose prior to the arrival of EMS, patients still require a thorough medical history and examination to assess the reasons for the overdose and potential sequelae.

Naloxone is beneficial prior to cardiac arrest, and of uncertain benefit during cardiac arrest. If naloxone has already been administered and the patient is not in cardiac arrest, EMS should ventilate the patient while waiting for effect. If EMS is administering naloxone, it is important to encourage appropriate dosing and titration to prevent withdrawal. The goal of naloxone therapy should be to reverse respiratory depression, not necessarily to awaken the patient to consciousness. Naloxone itself has mostly mild adverse effects but will precipitate acute withdrawal in opioid-dependent patients if not carefully titrated. This is an avoidable harm to patients and may reduce their willingness to further engage with care. Naloxone dosing remains a balancing act between onset of action and over-antagonism, and there are ongoing debates regarding dosing strategies for naloxone and other opioid antagonists, such as nalmefene. As the illicit drug supply evolves, dosing and other antidotes may become increasingly relevant with more potent opioids and other non-opioid substances affecting patients.

This link addresses gaps in prehospital emergency care and further follow-up. Patients experiencing hypoxia would typically be admitted to the hospital; however, many patients are left at the scene following overdose.^29^ Once again, the prevention of acute withdrawal is important to enable patients to engage in follow-up. EMS training in nonjudgmental and empathetic communication styles may decrease patients’ withdrawal-associated fear or anger and facilitate patients’ willingness to continue engaging in care.^30^

### Post-overdose care

The post-resuscitation period is an important opportunity to build rapport and initiate long-term interventions. From EMS transport onwards, this involves establishing pathways for assessment and initiation of treatment for the underlying causes of overdose. While an observation period following an opioid overdose and naloxone reversal is important, additional interventions could include early management of co-occurring issues, such as medical and mental health issues, substance use disorders, and social needs.^31^ Some may also benefit from assistance dealing with psychological, financial, and interpersonal survivorship sequelae. Harm reduction can also be emphasized when managing individual patient behaviors, such as desire for immediate re-dosing, leaving medical care prematurely, or using substances alone.

### Recovery and secondary prevention

The final link includes engaging the patient in the process of long-term aftercare for the issues identified in the prior link, as well as addressing risk factors for repeat overdose. In the case of opioid use disorder, this may include the provision of take-home naloxone, low-threshold induction of methadone or buprenorphine, discussion with peer recovery coaches, education regarding community resources, and referral to long-term addiction treatment. These interventions need not take place only in the hospital, as some innovative EMS agencies are performing buprenorphine induction after naloxone reversal.^32^ This may also include promoting overdose safety plans and education on harm reduction strategies. While more patients may be screened and initiated in treatment via implementation of the overdose chain of survival, it is important to develop sufficient community resources to effectively coordinate and provide care.

## Discussion

Drug overdose is a critical cause of preventable mortality from cardiac arrest. As a framework, the overdose chain of survival fills critical overdose-specific gaps in the AHA chain of survival formulations and in the continuity of overdose management. The overdose chain of survival can be useful in the treatment of individual patients, developing local care pathways, identifying research priorities and relevant outcomes, developing novel link-specific interventions, drafting overdose-related policy, and developing overdose-related educational initiatives. The overdose chain of survival can also support more nuanced systems mapping approaches to characterizing overdose events such as sequential intercept maps, causal loop diagrams, and decision tree models.^33^, ^34^, ^35^ This may enhance opportunities to identify specific points in the chain for public health intervention.

Although the cardiac arrest chain of survival framework can be applied to overdose, there are important differences between drug overdose and cardiac arrest. Overdose, especially from opioids, is often relatively simpler to acutely manage insofar as safe and effective antidotes exist. Moreover, the incidence of non-fatal overdose far exceeds that of survival from cardiac arrest, and many individuals who survive an overdose go on to experience subsequent overdoses, both fatal and non-fatal.^36^, ^37^ Unlike after cardiac arrest, overdose patients often decline further engagement with health services after initial resuscitation.^38^ This represents a challenging break in the chain involving complex discussions around patient autonomy, capacity and paternalism, as well as the risk of continued substance use and recurrent overdoses.^39^, ^40^, ^41^

Further investigation is needed to develop this framework. Additional research is also needed to optimize outcomes within each link, and to effectively coordinate care between links. The framework can be updated as the evidence base advances, the illicit drug supply changes, and overdose-related trends evolve.

## Conclusion

The proposed overdose chain of survival represents a structured framework to address cardiac arrest caused by drug overdose. By linking immediate overdose response with sustained recovery and prevention, it underscores the necessity for multidisciplinary and longitudinal care. As a major public health issue, overdose management requires cooperation and coordination across all levels of society, including bystanders, emergency services outside of and inside hospitals, and provision of longitudinal outpatient aftercare and community involvement. We encourage further refinement, implementation, and evaluation of this concept across clinical, public health, and policy settings to enhance outcomes and inform a more comprehensive response to the overdose crisis.

## Funding/disclosures

Nabarun Dasgupta is an uncompensated board member of the non-profit community-based organization Remedy Alliance For The People, which distributes bulk naloxone free or at-cost to government health departments and NGOs.

All other authors have no funding sources, disclosures, or potential conflicts of interest pertaining to this submission.

## Author agreement

All authors have read and agreed to the published version of the manuscript.

## Statement of adherence to preprint policy

This manuscript has not been posted on a preprint server.

## Statement of artificial intelligence use

The authors have not utilized any artificial intelligence (AI) in the creation of this manuscript.

## CRediT authorship contribution statement

**Matthew Robert Dernbach:** Writing – original draft, Project administration, Conceptualization. **Nabarun Dasgupta:** Writing – original draft, Conceptualization. **Adams L. Sibley:** Writing – original draft, Conceptualization. **Ida Tylleskar:** Writing – original draft, Conceptualization. **Arne Kristian Skulberg:** Writing – original draft, Project administration, Conceptualization.

## Declaration of competing interest

The authors declare that they have no known competing financial interests or personal relationships that could have appeared to influence the work reported in this paper.
